# Clinical characteristics of lacrimal drainage pathway disease-associated keratopathy

**DOI:** 10.1186/s12886-022-02580-y

**Published:** 2022-08-31

**Authors:** Hidenori Inoue, Koji Toriyama, Wakako Ikegawa, Yukako Hiramatsu, Arisa Mitani, Yuki Takezawa, Yuri Sakane, Tomoyuki Kamao, Yuko Hara, Atsushi Shiraishi

**Affiliations:** 1 Department of Ophthalmology, Ehime University Graduate School of Medicine, Shitsukawa, Toon, Ehime 791-0295 Japan; 2 Department of Ophthalmology, Matsuyama Red Cross Hospital, Bunkyocho, Matsuyama, Ehime 790-8524 Japan

**Keywords:** Keratopathy, Corneal ulcer, Corneal perforation, Chronic dacryocystitis, Lacrimal canaliculitis

## Abstract

**Purpose:**

To report the clinical characteristics of 13 cases of noninfectious corneal ulceration related to lacrimal drainage pathway disease.

**Methods:**

Medical records of 13 patients with lacrimal drainage pathway disease-associated keratopathy who were examined at Ehime University Hospital between April 2007 and December 2021 were analyzed.

**Results:**

The predisposing lacrimal drainage pathway diseases for corneal ulceration were chronic dacryocystitis in seven patients and lacrimal canaliculitis in six patients. The corneal ulcers were located at the peripheral cornea in 10 patients and the paracentral cornea in three patients. All patients indicated few cellular infiltrations of the ulcerated area at the slit-lamp examination. Corneal perforation was found in seven patients. The primary identified organisms were *Streptococcus spp.* in chronic dacryocystitis and *Actinomycetes spp.* in lacrimal canaliculitis. All patients showed rapid healing of the epithelial defects after treatment of the lacrimal drainage pathway disease. The mean time elapsed between treatment of the lacrimal drainage pathway disease and re-epithelialization of corneal ulcer was 14.5 ± 4.8 days.

**Conclusion:**

Lacrimal drainage pathway disease-associated keratopathy may be characterized by peripheral corneal ulcer with few cellular infiltrations, occasionally leading to corneal perforation. Treatment of the lacrimal drainage pathway disease could be the most effective treatment for lacrimal drainage pathway disease-associated keratopathy.

## Background

Chronic dacryocystitis is an infectious disease of the lacrimal drainage pathway that is caused by the obstruction or narrowing of the nasolacrimal duct. The consequent accumulation of lacrimal fluid and lacrimal sac contents leads to infection. The infection can be classified as acute or chronic dacryocystitis and is characterized by redness and swelling of the lacrimal sac and surrounding areas, conjunctival hyperemia, excessive tear flow, and copious ocular discharge.

Although the exact mechanism of lacrimal canaliculitis is not well characterized, it is believed to be triggered by a disorder of the lacrimal canalicular mucosa which results in infection of the lacrimal canaliculi and the formation of bacterial calculus. Lacrimal canaliculitis is common in older women and is typically unilateral [1]. The main complaint is excessive tear flow, large amounts of ocular discharge, and conjunctival hyperemia accompanied by redness and swelling of the lacrimal punctum. The condition is generally treated as intractable conjunctivitis.

Prospective studies have reported the relationship between nasolacrimal duct obstruction and infectious keratitis in several cases [2]. However, few reports propose that chronic dacryocystitis and lacrimal canaliculitis can induce noninfectious corneal ulcers. Most of the pertinent published literature comprises of individual case reports [3–8], and none of these reports have described the clinical characteristics of corneal ulcers. In this report, we describe 13 cases of noninfectious corneal ulcer related to lacrimal drainage pathway disease and assess the clinical characteristics. We refer to this disease as lacrimal drainage pathway disease-associated keratopathy (LDAK).

## Materials and methods

Clinical records of patients who were hospitalized for LDAK at the Ehime University Hospital between April 2007 and December 2021 were retrospectively reviewed. The demographic and clinical characteristics of patients including age, sex, systemic and ocular medical history, and systemic and local predisposing factors were collected. In systemic predisposing factors, seven cases were investigated for autoimmune diseases, including rheumatoid arthritis and Sjögren’s syndrome, by a laboratory test. In contrast, the others had validated by only a medical questionnaire. In addition, the characteristics of the corneal ulcer, viz., the location and shape, presence of epithelial defects, cellular infiltrations, corneal perforation, causative organism of the lacrimal drainage pathway disease, treatment details, and ulcer healing period were reviewed. The location of the ulcers was classified as peripheral (outer 1/2 of the cornea) and paracentral (inner 1/2 of the cornea). Ulcers in the peripheral area were classified as upper, inferior, nasal, and temporal at 90° from the center of the cornea. The causative organism was identified from culture of the lacrimal discharge performed at the time of diagnosis of the lacrimal drainage pathway disease. The discharge was incubated on sheep blood agar and Sabouraud agar at 37 °C. Bacterial identification was based on Gram staining and biochemical tests. The treatment methods for lacrimal drainage pathway disease, topical treatment, systemic administration, surgical intervention, and use of therapeutic contact lenses were also reviewed.

## Results

### Predisposing factors

The mean age of the 13 patients (6 male and 7 female) was 79.5 ± 7.2 years (range 68–91 years). The causative lacrimal drainage pathway disease was chronic dacryocystitis in 7 patients and lacrimal canaliculitis in 6 patients. The most common ocular predisposing factor was dry eyes in four patients (30.8%) and band keratopathy in one patient (7.7%). Among the systemic predisposing factors, three cases each (23.1%) had Sjögren’s syndrome and diabetes mellitus, while one patient (7.7%) had rheumatoid arthritis (Table 1).

**Table 1:** Characteristics of patients

	**Case no**	**Age (years)**	**Sex**	**Location of CU**	**Corneal Epithelial Defect**	**Corneal Infiltration**	**Corneal Perforation**	**Risk factors**	
								**Systemic**	**Ocular**
**Chronic dacryocystitis**	**1**	91	F	Paracentral	( +)	(-)	(-)	SS	Dry eye BK
	**2**	86	M	Inferior periphery	( +)	(-)	( +)	(-)	(-)
	**3**	82	F	Inferior periphery	( +)	(-)	(-)	DM	Dry eye
	**4**	77	M	Inferior periphery	( +)	(-)	(-)	DM	(-)
	**5**	85	M	Paracentral	( +)	(-)	( +)	(-)	(-)
	**6**	87	M	Superior periphery	( +)	(-)	(-)	RA	(-)
	**7**	81	M	Inferior periphery	( +)	(-)	( +)	SS DM	Dry eye
**Lacrimal canaliculitis**	**8**	71	M	nasal periphery	( +)	(-)	( +)	(-)	(-)
	**9**	68	F	Inferior periphery	( +)	(-)	( +)	SS	Dry eye PKP
	**10**	78	F	nasal periphery	( +)	(-)	(-)	(-)	(-)
	**11**	74	F	nasal periphery	( +)	(-)	( +)	(-)	(-)
	**12**	68	F	nasal periphery	( +)	(-)	(-)	(-)	(-)
	**13**	85	F	Paracentral	( +)	(-)	( +)	(-)	(-)

*M* Male, *F* Female, *CU* Corneal ulcer, *SS* Sjogren’s syndrome, *DM* Diabetes mellitus, *RA* Rheumatoid arthritis, *BK* Band keratopathy, *PKP* Penetrating keratoplasty

### Clinical findings

**Fig. 1:**
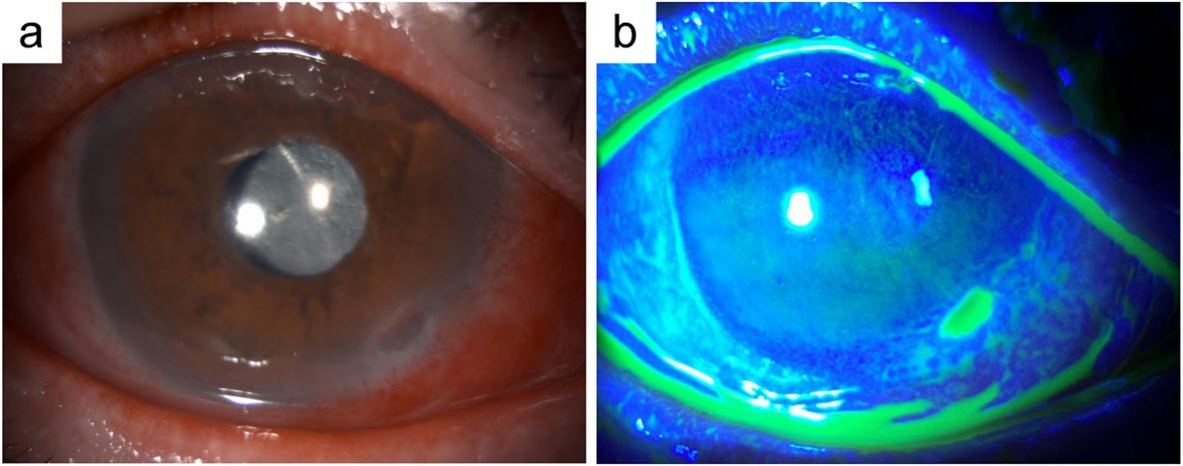
Representative slit-lamp photographs of lacrimal drainage pathway disease-associated keratopathy (LDAK). **a** Slit-lamp photograph shows the chronic dacryocystitis-associated corneal ulcer with few cellular infiltrations in the inferior peripheral cornea. **b** Photograph of the eye with fluorescein staining

The location of the corneal ulcer was peripheral in 10 patients (76.9%) and paracentral in three patients (23.1%). Of the 10 cases with peripheral lesions, five (50.0%) were inferior and four (40.0%) were nasal. All cases indicated few cellular infiltrations of the ulcerated area at slit-lamp examination (Table 1, Fig. 1). Seven patients developed corneal perforation during the clinical course, including four patients with lacrimal canaliculitis and three patients with chronic dacryocystitis.

### Identified organisms

In seven cases of chronic dacryocystitis, *Streptococcus spp.* was identified in four cases and *Staphylococcus spp.* was identified in three cases, one of which was methicillin-resistant *S. aureus* (MRSA). The other species identified were *Corynebacerium spp.*, *Pseudomonas aeruginosa*, *Klebsiella oxytoca*, and *Hemophilus influenzae*.

*Actinomyces spp.* were identified in three cases of lacrimal canaliculitis. Other species identified in the cases of lacrimal canaliculitis were *Streptococcus pneumoniae, Staphylococcus epidermidis, Enterococcus faecalis, Klebsiella pneumoniae, and Corynebacterium spp.*

More than one species were identified from a single specimen in some cases.

### Treatment details

All seven patients with chronic dacryocystitis underwent frequent lacrimal sac lavage, and five patients also underwent endoscopic-assisted nasolacrimal duct intubation (ENDI). The six patients with lacrimal canaliculitis underwent removal of the concretions in the lacrimal canal and one patient underwent canaliculotomy. Three patients also underwent ENDI (Table 2).

**Table 2:** Clinical course of patients

	**Case no**	**Treatment**			**Period from the diagnosis of LDAK to re-epithelialization (days)**
		**Before the diagnosis of LDAK**	**After the diagnosis of LDAK**		
			**Lacrimal drainage pathway disease**	**Other**	
**Chronic dacryocystitis**	**1**	(-)	ENDI Lacrimal sac lavage	Topical antimicrobial agents SCL	20
	**2**	(-)	Lacrimal sac lavage	Topical antimicrobial agents SCL	12
	**3**	Topical and systemic antimicrobial agents	Lacrimal sac lavage	Topical antimicrobial agents Topical steroids SCL	7
	**4**	Topical and systemic antimicrobial agents	ENDI Lacrimal sac lavage	Topical antimicrobial agents SCL	10
	**5**	(-)	ENDI Lacrimal sac lavage	Topical antimicrobial agents SCL	14
	**6**	Topical antimicrobial agents Topical steroids	ENDI Lacrimal sac lavage	Topical antimicrobial agents SCL	14
	**7**	Topical antimicrobial agents	ENDI Lacrimal sac lavage	Topical antimicrobial agents SCL	14
**Lacrimal canaliculitis**	**8**	Topical antimicrobial agents Topical steroids	Removal of concretions	Topical antimicrobial agents Topical steroids LKP SCL	23
	**9**	Topical antimicrobial agents Topical and systemic steroids PKP SCL	Removal of concretions	Topical antimicrobial agents Topical steroids SCL	21
	**10**	Topical antimicrobial agents Topical and systemic steroids	Removal of concretions ENDI	Topical antimicrobial agents Topical steroids SCL	18
	**11**	Topical antimicrobial agents Topical steroids	Removal of concretions canaliculotomy	Topical antimicrobial agents Topical steroids SCL	12
	**12**	(-)	Removal of concretions ENDI	Topical antimicrobial agents Topical steroids	7
	**13**	Topical antimicrobial agents	Removal of concretions ENDI	Topical antimicrobial agents Topical steroids SCL	16

*LDAK* Lacrimal drainage pathway disease-associated keratopathy, *SCL* Soft contact lens, *ENDI* Endoscopic-assisted nasolacrimal duct intubation, *LKP* Lamellar keratoplasty, *PKP* Penetrating keratoplasty

In all cases, topical quinolone (levofloxacin 1.5% or gatifloxacin 0.3% or moxifloxacin 0.5%) or cephem (cefmenoxime 0.5%) was used, either alone or in combination. Vancomycin 1.0% eye drops were used in the sole case with MRSA infection. Among the six cases of lacrimal canaliculitis, topical betamethasone 0.1% was used in one case and fluorometholone 0.1% in five cases. In two cases of chronic dacryocystitis, topical fluorometholone 0.1% was used. Therapeutic contact lenses were used concomitantly in 12 cases.

Seven patients developed corneal perforation during the follow-up period, and only one of these patients required corneal transplantation because the perforation site could not be closed with conservative treatment.

The period required for re-epithelialization of the corneal ulcers was examined as an indicator of the healing period. The healing period was divided into 2 periods; before LDAK diagnosis and after treatment of the primary lacrimal drainage pathway disease. The mean period from the onset of corneal ulcer to the diagnosis of LDAK was 23.3 ± 15.9 days. During this period, the corneal ulcer showed no improvement despite treatment with topical antibacterial and steroid therapy. After the diagnosis of LDAK, the mean period from the start of the treatment for lacrimal drainage pathway disease to corneal ulcer re-epithelialization was 14.5 ± 4.8 days. The healing period of the corneal ulcers was comparable in patients with chronic dacryocystitis and lacrimal canaliculitis (13.0 days and 16.2 days, respectively). None of the 13 patients developed recurrence of corneal ulcers after treatment of the lacrimal drainage pathway disease.

### Case report

**Fig. 2:**
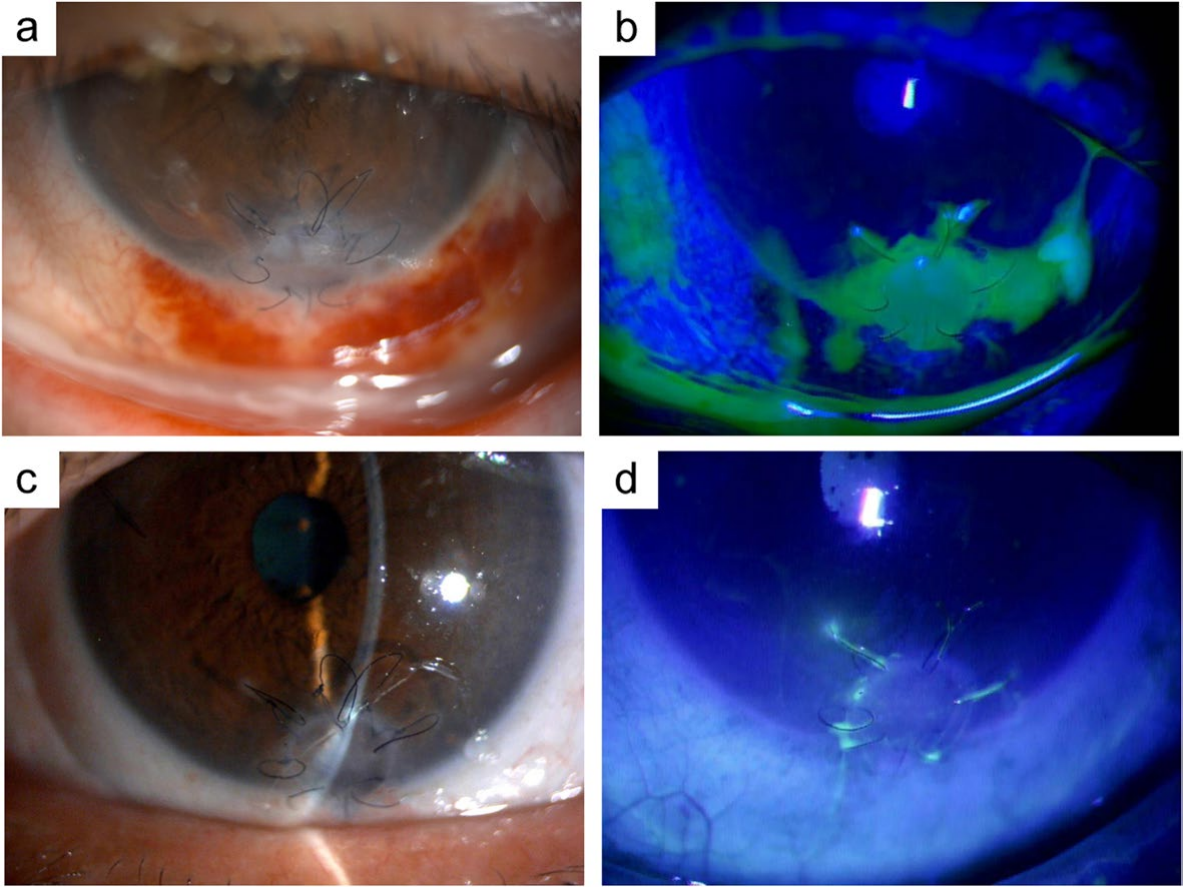
Slit-lamp photographs of a case of LDAK. **a**, **b** Corneal graft is melting with no signs of cellular infiltrations. **c**, **d** Corneal graft is re-epithelialized after the treatment of lacrimal drainage pathway disease

We present our findings in a representative case. A 68-year-old woman had dry eye due to Sjögren’s syndrome. She developed an unexplained melting of the lower periphery of the left cornea, which led to corneal perforation. The patient underwent penetrating keratoplasty (PKP) using preserved cornea at another hospital. However, despite the topical administration of antimicrobial agents and steroids and use of therapeutic contact lenses, the corneal graft melted immediately. Thus, the patient was referred to our hospital. At the first visit, copious ocular discharge and conjunctival hyperemia were observed, and the corneal graft was melting with no signs of cellular infiltrations. In addition, there was erythema and swelling of the left inferior lachrymal punctum. Dacryoendoscopy revealed lacrimal canaliculitis of the left inferior canaliculi. The patient underwent removal of concretions in the lacrimal canal. *Actinomyces spp.* was identified in the culture of the pus including the concretions. After the treatment of the lacrimal canaliculitis, the patient wore a therapeutic contact lens and used antibacterial and steroid eye drops. The corneal graft melting showed gradual improvement, and the cornea was re-epithelialized three weeks after the treatment for lacrimal canaliculitis. During the two-year follow-up, the patient had no recurrence of the corneal graft melting (Fig. 2).

## Discussion

We described 13 cases of noninfectious corneal ulcers caused by underlying lacrimal drainage pathway disease. We refer to this clinical condition as LDAK and have summarized the clinical characteristics. To the best of our knowledge, this is the largest case-series of LDAK.

In general, corneal ulcers can be divided into infectious and immune-mediated ulcers. Infectious corneal ulcers can present with different clinical features depending on the causative pathogen. Immune-mediated corneal ulcers typically present with peripheral corneal ulcers, represented by rheumatoid corneal ulcers and Mooren’s ulcers. Both infectious and immune-mediated ulcers are characterized by cellular infiltrations at the site of ulceration, reflecting corneal inflammation. Our cases of LDAK were characterized by nasal or inferior peripheral location of ulcers and copious ocular discharge. The most important feature of LDAK was few cellular infiltrations at the site of corneal ulceration. All of the 13 cases in our study showed few cellular infiltrations of the ulcers, which suggests that the ulcers in LDAK are not caused by corneal inflammation. In this study, a culture of the corneal ulcer site was conducted in one case of lacrimal canaliculitis; however, the causative organism was not detected. Cohn et al. reported one case of corneal ulcer associated with chronic dacryocystitis in which the bases of the ulcer were free of infiltrating, and bacteria were detected from the lacrimal sac but not from the peripheral corneal ulcers [3]. This is consistent with our review and is suggestive of the lack of infection at the cornea.

We also reviewed microbial isolates from the lacrimal drainage pathway at initial examination to investigate whether any particular causative organism of lacrimal drainage pathway disease causes LDAK. In the cases of chronic dacryocystitis, *Streptococcus spp.* and *Staphylococcus spp.* were dominantly identified. These species are consistent with the previously reported causative organisms of chronic dacryocystitis [9]. *Actinomyces spp.* were mainly detected in cases of lacrimal canaliculitis. The previously reported primary causative organisms of lacrimal canaliculitis are anaerobic bacteria such as *Actinomyces spp*., *Streptococcus spp*., and *Staphylococcus spp* [1]. There was no difference between the common causative organisms of chronic dacryocystitis or lacrimal canaliculaitis and that of LDAK. In the present review, *Actinomyces spp.* was identified in many cases of lacrimal canaliculitis. *Actinomyces* forms proteolytic enzymes, which may be associated with corneal ulceration [6]. Some reports suggest that LDAK is possibly induced by toxins from the bacteria that cause the lacrimal drainage pathway disease [3–6]. The relationship between the causative agent of lacrimal drainage pathway disease and LDAK needs to be investigated in a larger cohort.

In our study, the corneal ulceration healed quickly after the treatment of the lacrimal drainage pathway disease in all cases, which is consistent with previous reports [3, 4, 6]. These results suggest that lacrimal drainage pathway disease is an essential factor for the development of corneal ulceration. In previous reports about LDAK, good outcomes have been obtained with the treatment of lacrimal drainage pathway disease with or without steroids [3, 4, 6]. In our study, there was no significant difference in the duration of treatment period irrespective of the use of steroids (with steroids:10 ± 6 days, without steroids:15 ± 3 days, *P* = 0.87, Mann–Whitney *U* test). Because LDAK is presumed to be not caused by infection of the cornea but is related to an immune response in the lacrimal drainage pathway, the use of steroids may be effective in suppressing the immune response. Although no significant difference was discovered in the present number of cases, most cases treated with steroids were lacrimal canaliculitis (Table 2). Because predisposing diseases for LDAK differed significantly between cases treated with and without steroids, there is a possibility that these differences affected the treatment period. More studies with many cases are crucial to assess the effectiveness of steroids for treating LDAK.

Previous reports have described cases of corneal perforation induced by either chronic dacryocystitis or lacrimal canaliculitis [4–8]. Three of our patients with chronic dacryocystitis and four patients with lacrimal canaliculitis developed corneal perforation. Although no study has compared the characteristics of corneal ulcers caused by chronic dacryocystitis and lacrimal canaliculitis, there was no apparent difference in the shape of the ulcers including the rate of corneal perforation between the two pathological conditions in our series. With respect to the treatment of corneal perforation accompanying LDAK, Yokogawa et al. reported two patients who required surgical treatment, one of whom was treated with peripheral anterior lamellar keratoplasty and the other with multilayered amniotic membrane transplantation; both patients showed good treatment outcomes [5]. Ucar et al. performed conjunctival autografting [7]. In both reports, treatment of lacrimal drainage pathway disease was combined with surgical intervention for corneal perforation [5, 7]. On the other hand, Komatsu et al. and Ishikawa et al. reported that the corneal perforation can be closed by contact lenses in addition to treatment of the underlying lacrimal drainage pathway disease [4, 6]. In our series, surgical treatment was required only in one out of the seven cases with corneal perforation. In the other six cases, the perforation site was closed and re-epithelialized by wearing contact lenses after the treatment of lacrimal drainage pathway disease. Patients with lacrimal drainage pathway disease have been reported to show elevated levels of various inflammatory cytokines such as interleukins and matrix metalloproteinases in tear fluid [10, 11]. Wearing contact lenses can reduce the local turnover of tear fluid on the corneal surface [12] and possibly block these inflammatory cytokines. Thus, contact lenses may be beneficial for the treatment of LDAK by functioning as both mechanical and chemical barriers.

Some limitations of our study should be acknowledged. First, this was a relatively small retrospective, single-center case-series. More extensive studies are needed for in-depth characterization of the clinical features and proposing diagnostic criteria of LDAK. Second, the mechanisms underlying the development of LDAK remain unclear. Various factors in the tear fluid that has regurgitated from the lacrimal drainage pathway are supposed to be associated with ulcer formation. Future studies should include analysis of the tear fluid in LDAK to determine the relationship between the causative factors and the formation of corneal ulcers.

## Conclusions

Lacrimal drainage pathway diseases are a potential risk factor for corneal ulcer. The primary findings suggestive of LDAK are few cellular infiltrations at the site of ulceration and a large amount of discharge. Treatment of the primary lacrimal drainage pathway disease is the most effective treatment for LDAK. Although LDAK sometimes shows rapid progression to corneal perforation, it is not well recognized by general ophthalmologists or even corneal specialists. Clinicians should consider the possibility of lacrimal drainage pathway disease when encountering corneal ulcers with few cellular infiltrations but a large amount of discharge. Further clinical and experimental studies are required to unravel the mechanisms of ulcer formation.

## Data Availability

The datasets generated and/or analyzed during the current study are available from the corresponding author on reasonable request.

## Abbreviations

BK: Band Keratopathy
CU: Corneal ulcer
DM: Diabetes Mellitus
ENDI: Endoscopic-assisted Nasolacrimal Duct Intubation
F: Female
LDAK: Lacrimal drainage pathway Disease-Associated Keratopathy
LKP: Lamellar KeratoPlasty
MRSA: Methicillin-Resistant Staphylococcus Aureus
M: Male
PKP: Penetrating KeratoPlasty
RA: Rheumatoid Arthritis
SCL: Soft Contact Lens
SS: Sjögren’s Syndrome
